# Biopsychosocial determinants of tuberculosis care and treatment outcomes: a scoping review and bibliometric analysis

**DOI:** 10.3389/fcimb.2026.1958725

**Published:** 2026-09-24

**Authors:** Bayu Satria Wiratama, Susaldi Susaldi, Mohammad Hendra Setia Lesmana, M. Rizal Martua Damanik, Guntari Prasetya, Rathi Paramastri, Woro Rukmi Pratiwi, Ahmad Hamim Sadewa

**Affiliations:** 1 Department of Biostatistics, Epidemiology, and Population Health, Faculty of Medicine, Public Health, and Nursing, Universitas Gadjah Mada, Yogyakarta, Indonesia; 2 Faculty of Medicine, Public Health and Nursing, Universitas Gadjah Mada, Yogyakarta, Indonesia; 3 Faculty of Health Sciences, Universitas Indonesia Maju, Jakarta, Indonesia; 4 Department of Mental and Community Nursing, Faculty of Medicine, Public Health and Nursing, Universitas Gadjah Mada, Yogyakarta, Indonesia; 5 Faculty of Medicine and Nutrition, IPB University, Bogor, West Java, Indonesia; 6 Doctoral Study Program in Nutrition Science, Faculty of Medicine and Nutrition, IPB University, Bogor, West Java, Indonesia; 7 Department of Nutrition, STIKes Mitra Keluarga, Bekasi, West Java, Indonesia; 8 Department of Nutrition, Faculty of Public Health, Universitas Ahmad Dahlan, Yogyakarta, Indonesia; 9 Department of Pharmacology and Therapy, Faculty of Medicine, Public Health and Nursing, Universitas Gadjah Mada, Yogyakarta, Indonesia; 10 Department of Biochemistry, Faculty of Medicine, Public Health and Nursing, Universitas Gadjah Mada, Yogyakarta, Indonesia

**Keywords:** bibliometric analysis, biopsychosocial model, scoping review, social determinants of health, treatment outcomes, tuberculosis, tuberculosis care

## Abstract

**Background:**

Tuberculosis (TB) remains the leading infectious-disease cause of death globally, with an estimated 10.7 million incident cases and 1.23 million deaths in 2024. Its persistence is driven not by microbial factors alone, but by a complex interplay of biological, psychological, and social determinants influencing exposure, vulnerability, and access to care. Despite growing recognition of these biopsychosocial dimensions, the available evidence remains fragmented across disciplines, and no previous review has comprehensively mapped their scope while simultaneously characterizing the structure and evolution of this research field.

**Methods:**

We conducted an integrated scoping review and bibliometric analysis. Scopus, PubMed, and Web of Science were searched from database inception through 21 January 2026. Empirical studies examining biological, psychological, or social determinants associated with TB care or treatment outcomes were eligible. Data were extracted using a structured charting framework following Joanna Briggs Institute (JBI) methodology and reported according to the PRISMA Extension for Scoping Reviews (PRISMA-ScR). Bibliometric analyses were performed using VOSviewer and the Bibliometrix R package to characterize publication trends, collaboration networks, and thematic evolution. Study-specific effect estimates were summarized descriptively where available; meta-analysis was not undertaken because of substantial clinical and methodological heterogeneity.

**Results:**

A total of 124 studies were included, identifying 78 determinants across biological (n = 25), psychological (n = 25), and social (n = 28) domains. HIV co-infection, diabetes, malnutrition, smoking, alcohol use, stigma, limited TB knowledge, poverty, and barriers to healthcare access were consistently associated with delayed diagnosis, poor treatment adherence, multidrug-resistant TB (MDR-TB), unsuccessful treatment outcomes, and mortality. Bibliometric analysis demonstrated sustained growth in research output and an expansion from predominantly biomedical topics toward mental health, social determinants, digital health, and data-driven approaches. Integration of findings identified evidence gaps, with several determinants strongly associated with adverse outcomes receiving comparatively limited research attention.

**Conclusion:**

Integrating a scoping review with bibliometric analysis provides a comprehensive understanding of biopsychosocial determinants associated with TB care and treatment outcomes while identifying evidence gaps and emerging research priorities. These findings support multidisciplinary TB control strategies addressing biological, psychological, and social determinants to strengthen person-centered care.

**Systematic review registration:**

https://osf.io/8fpky/, identifier 8fpky.

## 1 Introduction

Tuberculosis (TB) remains a major global public health challenge, but its burden is far from evenly distributed. According to the World Health Organization (WHO), approximately 10.7 million people developed TB and 1.23 million died from the disease in 2024, making TB the leading cause of death from a single infectious agent worldwide (World Health Organization, 2024). The South-East Asia, Western Pacific, and African regions accounted for 34%, 27%, and 25% of incident cases, respectively. Eight countries, including India, Indonesia, the Philippines, China, Pakistan, Nigeria, the Democratic Republic of the Congo, and Bangladesh, contributed approximately two-thirds of the global burden (World Health Organization, 2024). Progress towards TB elimination has also remained slower than expected. Between 2015 and 2024, the global TB incidence rate declined by only 12.3%, well below the 50% reduction targeted under the WHO End TB Strategy for 2025 (Kurasawa, 2011; World Health Organization, 2024). These patterns suggest that the persistence of TB cannot be explained by microbial transmission or biomedical limitations alone. The disease is closely linked to unequal exposure, differences in biological vulnerability, limited access to resources, and the varying ability of individuals and communities to enter and remain within systems of care (Kurasawa, 2011; Jain and Dixit, 2012; Glaziou et al., 2015; Bian et al., 2024).

TB is increasingly recognized as a biopsychosocial condition shaped by the interaction of biological, psychological, social, and structural influences. Biological vulnerability may be affected by undernutrition, HIV infection, diabetes, age, sex, and other comorbid conditions that alter the risk of developing active disease or experiencing poor recovery (Kurasawa, 2011; Bian et al., 2024; World Health Organization, 2024). At the same time, fear, stigma, depression, treatment-related distress, limited understanding of the disease, and low confidence in healthcare services can influence symptom recognition, disclosure, care-seeking, and adherence (Dohál et al., 2023; Abaynew et al., 2025; Edward and Owoicho, 2025). These individual experiences occur within wider social conditions. Poverty, food insecurity, insecure employment, overcrowded housing, migration, inadequate social support, geographical barriers, and weak social protection can increase exposure to TB while also limiting access to continuous care (Harling et al., 2008; Jain and Dixit, 2012; Lima et al., 2015; Alagna et al., 2021; Bian et al., 2024; Suseendar et al., 2024; Edward and Owoicho, 2025; Pandey et al., 2025). Social characteristics, including education, occupation, gender, household composition, ethnicity, migration status, and place of residence, may further shape how these vulnerabilities are experienced. These domains often overlap, and their effects are rarely isolated in everyday life.

A biopsychosocial perspective is therefore essential for understanding why people may experience difficulties at different points in the TB care continuum. A person living with diabetes or undernutrition, for example, may also face stigma, unstable employment, transport costs, limited family support, and psychological distress. The combined effect of these conditions may delay care-seeking and diagnosis, hinder treatment initiation, or increase the risk of treatment interruption and loss to follow-up. Over time, they may contribute to treatment failure, recurrence, disability, or death (Harling et al., 2008; de Alencar Ximenes et al., 2009; Dohál et al., 2023; Abaynew et al., 2025; Dutta and Ranjan, 2025). The relationship also works in the opposite direction. TB can lead to loss of income, interruption of employment, catastrophic expenditure, social exclusion, and deterioration in mental well-being, thereby worsening the conditions that already make care difficult (Harling et al., 2008; de Alencar Ximenes et al., 2009; Abaynew et al., 2025). Studying each determinant separately may therefore overlook cumulative disadvantage and fail to explain how structural inequalities are translated into individual experiences and clinical outcomes. An integrated framework provides a stronger basis for identifying modifiable points of intervention and for designing responses that include psychological support, nutritional assistance, social protection, community engagement, and equitable health services (Harling et al., 2008; de Alencar Ximenes et al., 2009; Jain and Dixit, 2012; Glaziou et al., 2015; Bian et al., 2024; Dutta and Ranjan, 2025).

Despite increasing recognition of these relationships, the available evidence remains fragmented. Relevant studies are distributed across epidemiology, psychology, sociology, behavioral science, health-services research, and implementation science. They also differ in terminology, conceptual frameworks, measurement tools, study populations, geographical settings, and definitions of challenges and outcomes. Previous reviews have often concentrated on a single issue, such as poverty, stigma, mental health, gender, or adherence. Others have examined only one stage of care or a particular country or population. As a result, there is still no comprehensive picture of how biological, psychological, and social determinants operate across the broader TB care continuum. It also remains unclear which determinants, populations, outcomes, and regions have received the most research attention, and which remain overlooked. This fragmentation limits the usefulness of narrowly focused evidence synthesis for understanding the overall scope and structure of the field.

A scoping review is well suited to addressing this problem because it can map a broad and heterogeneous body of evidence, clarify how key concepts have been defined, and describe variations in study design, population, determinant domain, stage of care, and outcome (Pham et al., 2014; Munn et al., 2018). However, evidence mapping alone cannot fully show how the field has developed over time or how research activity is distributed across authors, institutions, countries, and thematic areas. Bibliometric analysis provides this additional perspective by examining publication growth, citation patterns, collaboration networks, keyword relationships, and the evolution of research themes (Arksey and O’Malley, 2005). Combining the two approaches allows the content of the evidence to be assessed alongside the structure of knowledge production. The scoping review can identify what is known about determinants and TB-related challenges, while the bibliometric analysis can show where that knowledge has been produced, how it is connected, and which topics remain underrepresented (Arksey and O’Malley, 2005; Donthu et al., 2021).

To our knowledge, previous global reviews have not integrated a comprehensive mapping of biopsychosocial determinants associated with tuberculosis (TB) care and clinical outcomes with a bibliometric assessment of the development and structure of this research field. This study therefore aimed to comprehensively map the biological, psychological, and social determinants associated with TB care and treatment outcomes through an integrated scoping review and bibliometric analysis. Specifically, we sought to identify and synthesize the determinants associated with TB care and treatment outcomes, characterize the evolution of this research field by examining publication trends, collaboration networks, and thematic development, and integrate findings from both approaches to identify evidence gaps and emerging research priorities that may inform future multidisciplinary research and person-centered TB care.

## 2 Methods

### 2.1 Study design and reporting framework

This study used an integrated evidence-synthesis design that combined a scoping review with bibliometric analysis to map the biopsychosocial determinants associated with challenges in tuberculosis care and outcomes worldwide. The scoping review was selected because the available evidence encompasses diverse study designs, populations, settings, determinant domains, and outcome measures. The review was conducted in accordance with the *Joanna Briggs Institute* methodology for scoping reviews and reported following the Preferred Reporting Items for Systematic Reviews and Meta-Analyses Extension for Scoping Reviews, or PRISMA-ScR, to support methodological transparency and reproducibility (Tricco et al., 2018; Peters et al., 2020).

The study was conducted through four sequential stages. First, the review objectives, eligibility criteria, and conceptual scope were defined. Second, relevant studies were systematically identified, duplicates were removed, eligible publications were selected through title, abstract, and full-text screening, and data were charted for descriptive evidence mapping. Third, bibliographic metadata from the included publications were extracted, standardized, and analyzed to examine publication trends, thematic structures, and patterns of collaboration. Finally, findings from the scoping review and bibliometric analysis were integrated to provide a comprehensive synthesis of the biopsychosocial determinants associated with TB care and treatment outcomes, identify evidence gaps, and highlight emerging research priorities.

The scoping review provided a structured synthesis of the biological, psychological, and social determinants associated with TB care and treatment outcomes identified in the literature. The bibliometric analysis complemented this evidence mapping by characterizing the development of the research field through publication trends, collaboration networks, and thematic evolution. Integrating these two approaches enabled the study to synthesize the available evidence, characterize the research landscape, and identify evidence gaps and emerging research priorities.

### 2.2 Data sources and search strategy

A comprehensive literature search was conducted in Scopus, PubMed, and Web of Science (WoS) to capture biomedical, public health, behavioral, and health-services research relevant to biopsychosocial determinants across the tuberculosis (TB) care continuum. Each database was searched from inception to its final search date, with all searches conducted between 26 December 2025 and 21 January 2026. Eligibility was restricted to peer-reviewed journal articles published in English to maintain consistency in screening, data charting, and interpretation. All retrieved records were exported in standardized formats for duplicate removal, screening, and subsequent analysis.

The search strategy was developed to identify studies examining TB in relation to biological, psychological/behavioral, and social determinants across the TB care continuum, including diagnosis, treatment initiation, adherence, continuity of care, and treatment outcomes. Boolean operators and database-specific field tags were applied. The complete database-specific search strategies, including the search terms, combinations, filters, and dates of execution, are provided in **Supplementary Material S1** to ensure transparency and reproducibility.

### 2.3 Eligibility criteria and study selection

Eligibility was operationalized using the population–concept–context framework together with predefined source-type criteria. The population comprised individuals or populations affected by TB, including people with presumptive or confirmed drug-susceptible or drug-resistant TB. Studies involving caregivers, healthcare providers, or other groups involved in TB care were eligible when the reported findings were directly related to patient care, care-seeking, treatment adherence, continuity of care, or treatment outcomes. The concept of interest was any biological, psychological/behavioral, or social determinant associated with a defined stage of the TB care continuum or with a treatment outcome. The context included all geographical, community, and healthcare settings.

Eligible sources were peer-reviewed English-language journal articles reporting original empirical data. Quantitative observational studies, including cross-sectional, case-control, and cohort studies, as well as intervention studies, qualitative studies, and mixed-methods studies, were eligible when they examined at least one relevant determinant in relation to TB care or treatment outcomes. Qualitative studies were considered relevant when they reported primary evidence concerning care-seeking, stigma, treatment experiences, adherence, barriers, facilitators, or continuity of care. Mixed-methods studies were eligible when at least one quantitative or qualitative component met the inclusion criteria; only findings relevant to the review objectives were subsequently charted and synthesized.

Studies were excluded if they did not focus on TB; did not examine a relevant biological, psychological/behavioral, or social determinant; or did not report a TB care-related or treatment outcome. Studies addressing exclusively diagnostic, laboratory, genomic, or pharmacological aspects were excluded when they did not establish an explicit relationship with TB care, treatment adherence, treatment success or failure, loss to follow-up, drug resistance, recurrence, or mortality. Reviews, meta-analyses, editorials, commentaries, protocols, conference abstracts, grey literature, and other publications that did not constitute eligible peer-reviewed original research articles were excluded. Studies combining TB with other diseases, conditions, or populations were also excluded when TB-specific findings could not be separately extracted.

Study selection followed a staged process consistent with PRISMA-ScR guidance. After duplicate removal, titles and abstracts were assessed against the predefined population, concept, context, and source-type criteria. Records were retained for full-text assessment when their relevance could not be confidently determined from the title and abstract alone. During full-text assessment, inclusion required an explicit relationship between at least one eligible determinant and a defined TB care-related or treatment outcome. Reasons for exclusion at the full-text stage were recorded using standardized categories and are presented in the PRISMA-ScR flow diagram (**Figure 1**). The final set of eligible studies constituted the scoping-review corpus for data charting and narrative synthesis.

**Figure 1:**
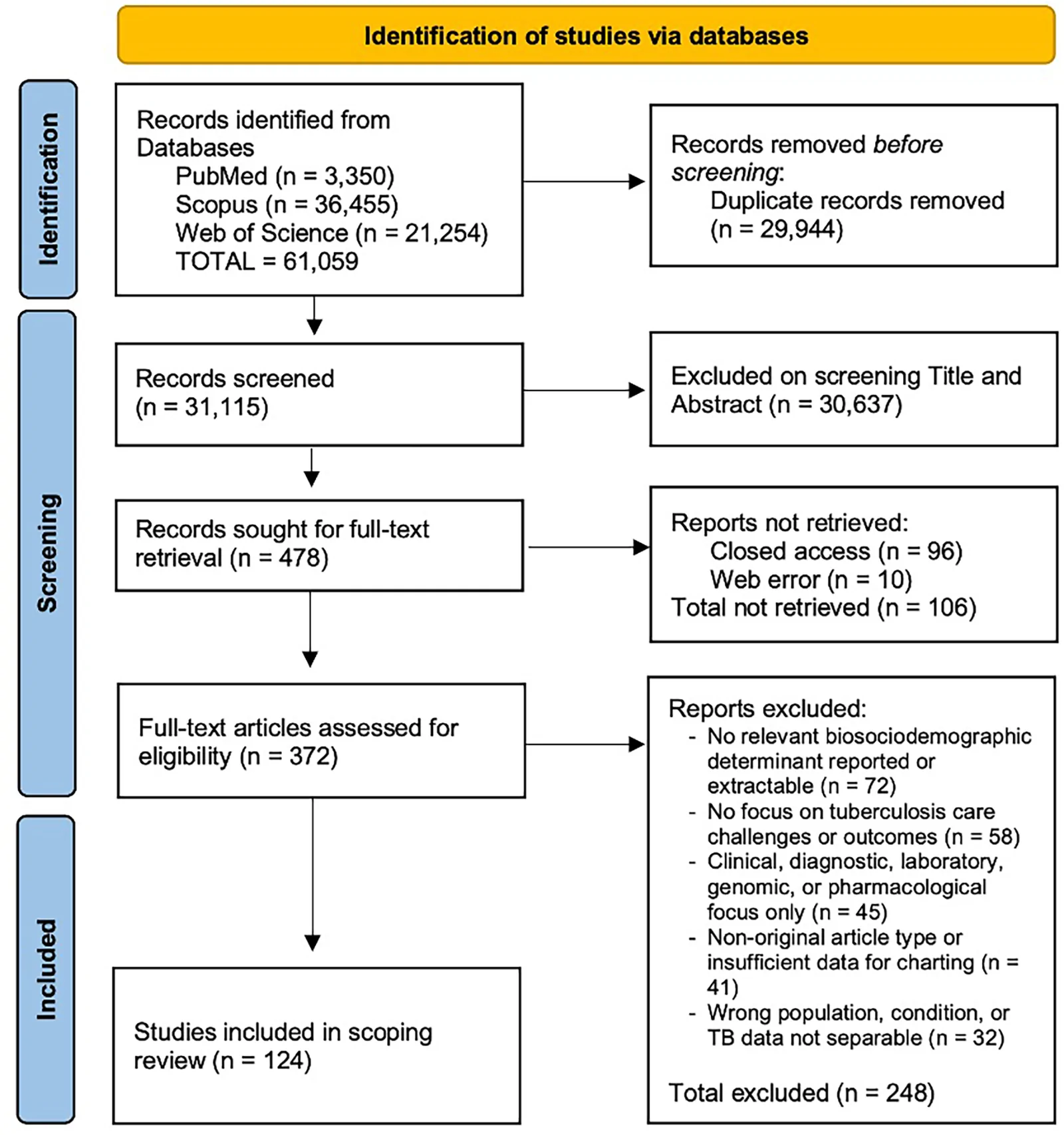
PRISMA flow diagram of the study selection process. A total of 61,059 records were identified from PubMed, Scopus, and Web of Science. After duplicate removal, records were screened by title and abstract, and 478 reports were sought for full-text retrieval. Of these, 106 reports were not retrieved because of closed access or web errors. The remaining 372 full-text reports were assessed for eligibility, with 248 excluded for reasons including absence of extractable biosociodemographic determinants, no focus on tuberculosis care challenges or outcomes, purely clinical/diagnostic/laboratory/genomic/pharmacological scope, non-original article type or insufficient data for charting, and wrong population/condition or non-separable tuberculosis data. Finally, 124 studies were included in the scoping review.

### 2.4 Data extraction, bibliometric analysis, and synthesis

Data extraction was undertaken through a structured charting process developed *a priori* to systematically capture information relevant to the objectives of this review. The charting framework was designed to accommodate variability in study designs and reporting practices while ensuring consistency across the extracted dataset (Arksey and O’Malley, 2005). Extracted information included bibliographic characteristics, study design, participant characteristics, geographical setting, biological determinants, psychological determinants (including psychosocial and behavioral factors), social determinants (including social factors), and reported TB care and treatment outcomes. The extraction process was refined iteratively to ensure that all relevant dimensions were consistently represented across the included studies (Peters et al., 2020).

Study-level characteristics charted for the descriptive summary included the primary study design and the country or geographical setting in which each study was conducted. Single-country studies were assigned to the corresponding World Health Organization region, whereas studies involving countries from more than one WHO region were classified as multicountry or global. These characteristics were subsequently summarized in **Table 1**. For mixed-methods studies, quantitative and qualitative findings relevant to the review objectives were charted separately. Quantitative findings were extracted together with their reported measures of association, whereas qualitative findings were coded according to the relevant determinant, stage of the TB care continuum, and outcome domain and were synthesized narratively. Qualitative findings were not converted into numerical effect estimates.

**Table 1:** Distribution of the included sources of evidence by primary study design and World Health Organization region (N = 124).

Dimension	Category	Sources of evidence, n	Percentage, %
**Primary study design**	Cohort or longitudinal study	39	31.5
	Cross-sectional study	38	30.6
	Registry-, surveillance-, or spatial-analysis study	18	14.5
	Other retrospective or observational study	16	12.9
	Case–control study	7	5.6
	Mixed-methods study	6	4.8
	**Total**	**124**	**100**
**WHO region**	African Region	43	34.7
	Western Pacific Region	32	25.8
	South-East Asia Region	15	12.1
	European Region	13	10.5
	Eastern Mediterranean Region	11	8.9
	Region of the Americas	10	8.1
	**Total**	**124**	**100**

Percentages were calculated using the 124 included sources of evidence as the denominator within each dimension. Study-design descriptions were harmonized into mutually exclusive categories based on the principal methodological approach reported for each source. Cohort or longitudinal studies included prospective, retrospective, registry-based cohort, and follow-up designs. Cross-sectional studies included analytic, institution-based, facility-based, and registry-based cross-sectional designs. Sequential explanatory designs and studies explicitly integrating quantitative and qualitative components were classified as mixed-methods studies. Studies based primarily on surveillance, registry, routine programmatic, geospatial, or spatial epidemiological data were grouped as registry-, surveillance-, or spatial-analysis studies. Studies for which the available description did not permit assignment to a more specific conventional design were categorized as other retrospective or observational studies. Countries were assigned to World Health Organization regions according to the location in which study data were collected. WHO, World Health Organization. Bold values indicate totals within each dimension.

Where reported, study-specific measures of association, including odds ratios, adjusted odds ratios, hazard ratios, adjusted hazard ratios, and risk or rate ratios, were charted together with the corresponding determinant and TB-related outcome. For determinants examined in multiple studies, estimates were presented as individual values or descriptive ranges when the effect measures, outcomes, and reference categories were sufficiently interpretable. No pooled effect estimates were calculated because the included studies differed substantially in design, population, determinant and outcome definitions, reference groups, follow-up periods, and covariate-adjustment strategies. Qualitative findings and quantitatively non-comparable results were synthesized according to the direction and context of the reported association.

Bibliometric analysis was subsequently conducted to characterize the development and structure of research on biopsychosocial determinants associated with tuberculosis (TB) care and clinical outcomes. Bibliographic metadata were standardized and harmonized before analysis to ensure consistency across databases. Science mapping and network analyses were performed using VOSviewer to visualize co-authorship networks, keyword co-occurrence, and thematic structures, while complementary bibliometric analyses were conducted in the RStudio environment using the Bibliometrix package (van Eck and Waltman, 2010; Aria and Cuccurullo, 2017). VOSviewer analyses were performed using bibliographic metadata retrieved from Scopus, PubMed, and Web of Science (WoS), whereas Bibliometrix analyses were conducted using metadata retrieved from PubMed only. This integrated analytical approach enabled the assessment of publication trends, country scientific production, collaboration networks, thematic evolution, and emerging research themes, complementing the evidence synthesis from the scoping review. For synthesis, findings from the scoping review and bibliometric analyses were integrated using a narrative and descriptive approach consistent with the objectives of evidence mapping rather than effect estimation. The scoping review findings were synthesized to describe the biological, psychological, and social determinants associated with TB care and treatment outcomes, while the bibliometric analysis was used to characterize publication trends, collaboration networks, and thematic evolution within the research field. Findings from both approaches were then integrated to identify areas of convergence, evidence gaps, and emerging research priorities.

## 3 Results

### 3.1 Study selection

The literature search identified 61,059 records from three electronic databases, including PubMed (n = 3,350), Scopus (n = 36,455), and Web of Science (n = 21,254) (**Figure 1**). After removing 29,944 duplicate records, 31,115 unique records remained for title and abstract screening. Of these, 30,637 records were excluded because they did not meet the eligibility criteria, leaving 478 articles for full-text retrieval. A total of 106 reports could not be retrieved owing to restricted access (n = 96) or web-related errors (n = 10), resulting in 372 full-text articles assessed for eligibility.

Among the full-text articles, 248 were excluded for the following reasons: absence of relevant biopsychosocial or social determinants (n = 72), failure to examine TB care challenges or clinical outcomes (n = 58), exclusive focus on clinical, diagnostic, laboratory, genomic, or pharmacological aspects (n = 45), lack of original data for data extraction (n = 41), or inclusion of populations or conditions that were not specific to TB (n = 32). Consequently, 124 studies met the eligibility criteria and were included in the scoping review (See **Figure 1**). Detailed determinant-level findings from the included studies are summarized in **Table 2**. The included evidence represented diverse study designs and geographical settings. The distribution of the included studies according to primary study design and geographical region is presented in **Table 1**.

**Table 2:** Determinants of tuberculosis risk and treatment outcomes across biological, sociodemographic, and psychosocial/behavioral domains.

Broad category	Specific determinant	Number of studies (N)	Direction of association	Key clinical/statistical findings
Biological	HIV co-infection	25	↑ Increased risk	Strongest predictor of mortality (aOR 2.2–4.85; aHR 1.67–4.21) and treatment failure (aOR 1.36–5.13); significantly associated with MDR-TB (aOR 1.9).
Biological	Male sex	22	↑ Increased risk	Independent predictor of unsuccessful outcomes (aOR 2.52) and mortality (HR 1.89–6.49); linked to treatment default (p=0.003) and non-adherence (OR 3.53).
Biological	Older age (e.g., > 45, > 65 years)	17	↑ Increased risk	Highest mortality in patients aged 80+ (aOR 70.80) and unsuccessful treatment (aOR 4.02); linked to diagnostic delay and comorbidity burden.
Biological	Low BMI (< 18.5 kg/m²)	10	↑ Increased risk	Predicts MDR-TB (aOR 3.9), mortality (aHR 4.13), and unsuccessful outcomes (aOR 2.86); associated with advanced radiographic disease.
Biological	Diabetes mellitus	8	↑ Increased risk	Predicts transmission risk (OR 1.40) and treatment failure (aOR 8.78); comprised ~25% of MDR-TB cases in some cohorts.
Biological	Drug resistance (MDR/XDR-TB)	6	↑ Increased risk	Strong predictor of unsuccessful treatment (aOR 6.44–9.32) and mortality (HR 3.35).
Biological	Female sex	5	↑ Increased risk	Associated with longer health-system delay (HR 0.63), diagnostic delay (aOR 2.04), and treatment abandonment.
Biological	Previous TB treatment (relapse/recurrent)	5	↑ Increased risk	Increased loss to follow-up (aOR 2.19) and mortality (HR 2.73); relapse cases showed 69% higher LTFU prevalence.
Biological	Malnutrition (MUAC < 12.5 cm / WAZ < -3)	4	↑ Increased risk	Associated with unsuccessful MDR-TB treatment (aOR 2.99) and pediatric mortality (OR 2.98).
Biological	Age 25–44 years	4	↑ Increased risk	Associated with loss to follow-up (aOR 2.83) and MDR-TB (aOR 3.8).
Biological	Age < 10 years (children/infants)	3	↑ Increased risk	Fourfold mortality risk (aHR 4.06); regionally associated with higher mortality (aOR 3.46).
Biological	Chronic kidney disease	2	↑ Increased risk	Higher mortality (aHR 3.62) and unfavorable outcomes (aOR 9.89 in younger cohorts).
Biological	Extrapulmonary TB (EPTB)	2	↑ Increased risk	Threefold higher mortality (aOR 3.00); median diagnostic delay of 130 days.
Biological	Malignancy (active cancer)	2	↑ Increased risk	Strong association with treatment-phase death (aHR 3.60) and diagnostic delay (aOR 1.80).
Biological	Tobacco use / smoking	2	↑ Increased risk	Independent predictor of treatment default (aOR 4.1) and loss to follow-up (aOR 1.96).
Biological	Alcoholism / alcohol abuse	2	↑ Increased risk	Associated with unsuccessful treatment (aOR 1.84) and ~18-fold higher risk of precocious death.
Biological	Ethnicity	2	↑ Increased risk	Miao (aOR 1.38) and Dong (aOR 1.88) show higher adverse-reaction risk; Hispanic (aRR 1.19) and Black (aRR 1.32) show higher treatment-related mortality.
Biological	Hemoptysis	2	↑ Increased risk	Associated with diagnostic delay > 3 weeks (aOR 25.37), though one source links it to shorter care-seeking delay.
Biological	Sputum smear positive	2	↑ Increased risk	High bacillary load (2+/3+) significantly reduced odds of treatment success (aOR 0.42).
Biological	Lung cavitation	2	↑ Increased risk	Strongly associated with poor MDR-TB outcomes; absence of cavitation raised odds of effective therapy 1.7-fold.
Biological	Hypoalbuminemia / low hemoglobin	1	↑ Increased risk	Predictive of in-hospital mortality (aOR 3.77 for albumin; aOR 3.34 for hemoglobin).
Biological	Psychological illness	1	↑ Increased risk	Strong association with MDR-TB (aOR 9.4); includes depression and anxiety.
Biological	Osteoporosis	1	↑ Increased risk	Independent risk factor for combined pulmonary/extrapulmonary TB (OR 4.93).
Biological	Autoimmune disease	1	↑ Increased risk	Associated with higher transmission risk (OR 2.23) among asymptomatic patients.
Biological	Liver disease	1	↑ Increased risk	Associated with higher hepatotoxicity risk from anti-TB medication (aOR 6.22).
Social	Age	15	Mixed / Context-dependent	Advancing age raises mortality, delay, and drug-resistance risk; younger age (infants/children/adolescents) shows elevated loss to follow-up or mortality in specific cohorts.
Social	Male gender	10	↑ Increased risk	Positively correlated with TB risk, unsuccessful outcomes, loss to follow-up, and mortality; higher notification incidence but occasionally shorter delay than women.
Social	Education level	8	Mixed / Context-dependent	Higher education lowers delay and improves treatment success; lower education/illiteracy raises drug resistance, non-adherence, and mortality risk.
Social	HIV status / coinfection	8	↑ Increased risk	HIV positivity significantly increases TB progression, unsuccessful treatment, mortality, and loss to follow-up.
Social	Travel distance to health facility	8	↑ Increased risk	Distance > 5 km is a major barrier, increasing treatment delay, non-adherence, and unsuccessful outcomes.
Social	Female gender	7	Mixed / Context-dependent	Often linked to better health-seeking behavior and treatment success, but also to diagnostic delay, non-adherence, and post-mortem notification in some contexts.
Social	Poverty / low income	7	↑ Increased risk	Low socioeconomic status and financial instability raise TB transmission, diagnostic delay, treatment interruption, and catastrophic health expenditure.
Social	Rural residence	6	Mixed / Context-dependent	Generally linked to poorer healthcare access, longer delay, and higher mortality, though some studies report no significant association or better adherence.
Social	Employment status	6	Mixed / Context-dependent	Unemployment/daily labor raises unsuccessful outcomes and non-adherence; demanding schedules among the employed can also cause missed doses and delay.
Social	Alcohol consumption	6	↑ Increased risk	Alcohol abuse/alcoholism raises mortality, treatment abandonment, drug-resistant TB, and adverse drug reactions.
Social	Smoking / tobacco use	6	↑ Increased risk	Tobacco use raises unsuccessful treatment outcomes, mortality, loss to follow-up, and MDR-TB development.
Social	Household crowding	5	↑ Increased risk	Overcrowded conditions (< 2 rooms or large families) raise transmission risk, infection risk, and diagnostic delay.
Social	Stigma	5	↑ Increased risk	Perceived social/family stigma raises patient delay, non-adherence, and MDR-TB risk.
Social	Homelessness	5	↑ Increased risk	Positively correlated with unsuccessful treatment, higher mortality, vulnerability, and prior TB infection.
Social	Migrant status	5	↑ Increased risk	Positively correlated with diagnostic delay, unsuccessful outcomes, financial hardship, and loss to follow-up.
Social	Low BMI / underweight	5	↑ Increased risk	Malnutrition and low BMI (< 18.5 kg/m²) raise risk of death, relapse, treatment failure, and MDR-TB.
Social	History of previous TB treatment	4	↑ Increased risk	Prior treatment failure or interruption is a primary driver of MDR-TB and future unsuccessful outcomes.
Social	Urban residence	4	↑ Increased risk	Urban/metropolitan settings show higher notification incidence, MDR-TB prevalence, and loss-to-follow-up risk.
Social	Lack of treatment supervision (DOTS)	4	↑ Increased risk	Absence of directly observed therapy raises treatment failure, loss to follow-up, and death risk.
Social	Imprisonment (prisoner status)	3	Mixed / Context-dependent	Confinement/overcrowding raises TB development and mortality risk, though diagnosis may be faster via routine prison screening.
Social	Marital status (unmarried/single)	3	↑ Increased risk	Unmarried, single, or divorced status raises loss to follow-up, poor adherence, and advanced disease presentation.
Social	Lack of medical insurance	3	↑ Increased risk	Functions as a financial barrier, increasing both patient delay and care-seeking delay.
Social	TB knowledge / health literacy	3	Mixed / Context-dependent	Limited knowledge of TB symptoms/treatment raises health-seeking delay, diagnostic delay, and drug-resistance risk.
Social	Treatment supporters	2	↓ Decreased risk (Protective)	Presence of a treatment supporter raises medication adherence and likelihood of successful outcome.
Social	Illicit drug use	2	↑ Increased risk	Positively correlated with pre-treatment loss to follow-up, treatment abandonment, and unfavorable outcomes.
Social	Type of health facility	2	Mixed / Context-dependent	Initial consultation at general hospitals/high-volume facilities often increases diagnostic delay versus public health centers.
Social	Ventilation	1	Mixed / Context-dependent	Poor ventilation (fewer windows or infrequent opening) raises MDR-TB risk.
Social	Occupational exposure (silica/dust)	1	↑ Increased risk	Silica dust exposure/silicosis raises TB development and mortality risk.
Psychological	Smoking / tobacco use	24	↑ Increased risk	Raises TB risk, mortality, treatment default, loss to follow-up, and MDR-TB; assessed via questionnaires, eCBSS, and GATS-India criteria.
Psychological	Alcohol use / alcoholism	23	↑ Increased risk	Raises TB risk, diagnostic delay, treatment default, mortality, and adverse drug reactions; assessed via DSM-IV, WHO AUDIT, and ICD-10 criteria.
Psychological	Knowledge of TB (awareness/literacy)	22	Mixed / Context-dependent	Limited knowledge/literacy raises diagnostic and health-seeking delay and non-adherence/MDR-TB risk; measured via KAP indexes and structured interviews.
Psychological	Stigma / perceived stigma	18	↑ Increased risk	Raises diagnostic delay, treatment interruption, and loss to follow-up; often drives non-disclosure. Measured via Likert scales and qualitative interviews (FGDs/KIIs).
Psychological	Treatment adherence / compliance	15	Mixed / Context-dependent	Poor adherence raises treatment failure, drug resistance, and mortality risk; assessed via DOT monitoring, MMAS-8, and pill counts.
Psychological	Illicit drug use / addiction	12	↑ Increased risk	Raises loss to follow-up, unsuccessful outcomes, MDR-TB, and mortality; captured via eCBSS and SINAN notification data.
Psychological	Previous TB treatment history	8	↑ Increased risk	Raises treatment failure, loss to follow-up, and MDR-TB development risk.
Psychological	Unemployment / poverty	8	↑ Increased risk	Raises diagnostic delay, TB risk, and non-adherence via financial barriers.
Psychological	Social / family support	8	↓ Decreased risk (Protective)	Presence of support raises adherence and treatment success; absence raises loss to follow-up and mortality risk.
Psychological	Distance to health facility	7	↑ Increased risk	Long travel time/distance raises diagnostic delay and non-adherence risk, with paradoxical findings in some contexts.
Psychological	HIV co-infection	7	↑ Increased risk	Raises mortality, diagnostic delay, and unsuccessful treatment risk.
Psychological	Migrant status	7	↑ Increased risk	Raises probability of diagnosis delay, treatment default, and unsuccessful outcomes.
Psychological	Educational level	6	Mixed / Context-dependent	Lower education raises mortality and diagnostic delay risk; university-level education lowers delay.
Psychological	Imprisonment (prisoners/inmates)	5	Mixed / Context-dependent	Raises TB risk and mortality but may lower diagnostic delay through routine prison screening.
Psychological	Khat chewing	4	↑ Increased risk	Raises MDR-TB, diagnostic delay, and treatment failure risk.
Psychological	Rural residence	4	↑ Increased risk	Raises risk of poor treatment outcomes and diagnostic delay.
Psychological	Malnutrition (low BMI)	2	↑ Increased risk	Raises unsuccessful treatment and mortality risk; assessed via MUAC/BMI measurement.
Psychological	Traditional / religious healers	2	↑ Increased risk	Initial consultation with traditional/religious healers raises diagnostic delay.
Psychological	Health-seeking behavior (informal care)	2	↑ Increased risk	Consulting non-formal providers/pharmacies first raises diagnostic delay.
Psychological	Patient attitude / beliefs	2	↑ Increased risk	Misconceptions and low perceived severity raise diagnostic delay; assessed via Health Belief Model frameworks.
Psychological	Psychological distress / depression	2	↑ Increased risk	Raises treatment non-adherence and MDR-TB risk; assessed via Kessler-10 (K-10) scale.
Psychological	Homelessness	2	↑ Increased risk	Raises unsuccessful treatment outcome and mortality risk.
Psychological	Direct observed therapy (DOT)	2	↓ Decreased risk (Protective)	Supervision (especially by relatives) raises treatment success; lack of DOT raises unfavorable-outcome risk.
Psychological	Employment status	2	Mixed / Context-dependent	Employment can paradoxically raise diagnostic delay (fear of income loss) or improve adherence (stable routine).
Psychological	Marital status (single/divorced)	2	↑ Increased risk	Raises loss-to-follow-up risk and reduces treatment success.

N denotes the number of studies reporting each determinant. Upward and downward arrows indicate increased and decreased risk, respectively, while “mixed/context-dependent” indicates variation in the direction of association across studies or settings. Effect estimates are reported only where available and represent study-specific adjusted or unadjusted estimates; reported ranges are descriptive and do not constitute pooled effects. Differences in study design, population, outcome definition, reference category, and covariate adjustment should be considered when interpreting estimates across studies. aOR, adjusted odds ratio; OR, odds ratio; HR, hazard ratio; aHR, adjusted hazard ratio; aRR, adjusted risk or rate ratio; MDR/XDR-TB, multidrug-/extensively drug-resistant tuberculosis; LTFU, loss to follow-up; DOTS/DOT, directly observed treatment/therapy.

### 3.2 Biopsychosocial determinants

This scoping review identified 78 determinants of tuberculosis (TB) risk and treatment outcomes across three thematic domains: biological (n = 25), psychological and behavioral (n = 25), and social (n = 28). The number of studies reporting each determinant ranged from one to 25, indicating substantial variation in the amount of available evidence. **Table 2** summarizes the number of contributing studies, direction of association, and key clinical or statistical findings for each determinant, including study-specific effect estimates or descriptive ranges where these were available.

#### 3.2.1 Biological determinants

Among the 25 biological determinants identified, immunological and infection-related factors were the most frequently reported. HIV co-infection was the most commonly reported determinant (n = 25) and was consistently associated with mortality, with adjusted odds ratios ranging from 2.2 to 4.85 and adjusted hazard ratios ranging from 1.67 to 4.21. HIV co-infection was also associated with multidrug-resistant TB (MDR-TB; aOR = 1.9). Chronic kidney disease (n = 2; aHR = 3.62), active malignancy (n = 2; aHR = 3.60), autoimmune disease (n = 1; OR = 2.23), and liver disease (n = 1; aOR = 6.22 for hepatotoxicity) were reported less frequently but were also associated with adverse outcomes.

Male sex was the second most frequently reported biological determinant (n = 22) and was associated with unsuccessful treatment outcomes (aOR = 2.52), mortality (HR = 1.89–6.49), treatment default (p = 0.003), and non-adherence (OR = 3.53). Older age (n = 17) was associated with mortality, with the highest reported estimate observed among individuals aged ≥80 years (aOR = 70.80). Age younger than 10 years (n = 3) was also associated with mortality (aHR = 4.06).

Low BMI (<18.5 kg/m²; n = 10) was associated with MDR-TB (aOR = 3.9), mortality (aHR = 4.13), and unsuccessful treatment outcomes (aOR = 2.86). Malnutrition (n = 4) was associated with unsuccessful MDR-TB treatment (aOR = 2.99) and pediatric mortality (OR = 2.98), while hypoalbuminemia (n = 1) was associated with mortality (aOR = 3.77). Diabetes mellitus (n = 8) was associated with TB transmission (OR = 1.40) and treatment failure (aOR = 8.78), and accounted for approximately one-quarter of MDR-TB cases in some studies.

Drug resistance (n = 6; aOR range = 6.44–9.32), extrapulmonary TB (n = 2), sputum smear positivity (n = 2), lung cavitation (n = 2), and hemoptysis (n = 2) were consistently associated with unsuccessful treatment outcomes or diagnostic delay. Few studies reported protective biological determinants. Because the included studies used different effect measures, outcome definitions, reference categories, and adjustment strategies across heterogeneous populations, quantitative pooling was not undertaken. Accordingly, the reported values and ranges represent study-specific estimates or descriptive ranges rather than pooled effects (**Table 2**).

#### 3.2.2 Psychological determinants

Smoking or tobacco use (n = 24) and alcohol use or alcoholism (n = 23) were the most frequently reported psychological determinants. Both were consistently associated with increased TB risk, mortality, treatment default, loss to follow-up, and MDR-TB. Illicit drug use or addiction (n = 12) and khat chewing (n = 4) were also associated with loss to follow-up, unsuccessful treatment, and MDR-TB.

Knowledge of TB (awareness or literacy) was the third most frequently reported determinant overall (n = 22). Limited TB knowledge was associated with diagnostic delay, delayed healthcare seeking, non-adherence, and MDR-TB. Similar findings were reported for lower educational attainment (n = 6) and patient attitudes or beliefs (n = 2).

Stigma or perceived stigma (n = 18) was associated with diagnostic delay, treatment interruption, loss to follow-up, and non-disclosure of TB status. Social and family support (n = 8) was associated with improved treatment adherence and treatment success, whereas lack of support was associated with loss to follow-up and mortality. Unemployment or poverty (n = 8), migrant status (n = 7), HIV co-infection (n = 7), homelessness (n = 2), and psychological distress or depression (n = 2) were also associated with adverse outcomes. Imprisonment (n = 5) was associated with increased TB risk and mortality, although some studies also reported shorter diagnostic delay among incarcerated populations.

Treatment adherence or compliance (n = 15) was associated with treatment outcomes, with poor adherence increasing the risk of treatment failure, drug resistance, and mortality. Directly observed therapy (DOT; n = 2) was associated with improved treatment outcomes. Previous TB treatment (n = 8), greater distance to healthcare facilities (n = 7), and the use of traditional or religious healers (n = 2 each) were associated with diagnostic delay or unsuccessful retreatment. HIV co-infection, smoking or tobacco use, alcohol use, low BMI, and previous TB treatment were identified across all three thematic domains, whereas age, sex, migrant status, education, employment, rural residence, and distance to healthcare facilities were identified in two domains.

#### 3.2.3 Social determinants

Among social determinants, age was the most frequently reported factor (n = 15). Older age was associated with mortality, delayed treatment, and drug resistance, whereas younger age groups were associated with loss to follow-up and mortality in several studies. Male gender (n = 10) was associated with TB risk, unsuccessful treatment outcomes, loss to follow-up, and mortality. Female gender (n = 7) was associated with improved healthcare seeking and treatment success in some studies but with diagnostic delay and non-adherence in others.

Education level (n = 8), poverty or low income (n = 7), stigma (n = 5), homelessness (n = 5), migrant status (n = 5), and low BMI or underweight status (n = 5) were associated with diagnostic delay, treatment interruption, catastrophic health expenditure, and adverse treatment outcomes. Employment status (n = 6) was also associated with treatment outcomes, with both unemployment and demanding work schedules reported as barriers to treatment adherence.

Health-system determinants included HIV co-infection (n = 8), distance to healthcare facilities (n = 8), previous TB treatment (n = 4), lack of directly observed treatment supervision (DOTS; n = 4), and lack of medical insurance (n = 3). Distances greater than 5 km were consistently associated with delayed treatment initiation.

Environmental determinants included household crowding (n = 5), rural residence (n = 6), urban residence (n = 4), imprisonment (n = 3), and occupational silica or dust exposure (n = 1). These factors were associated with TB transmission or adverse outcomes, although findings for rural residence varied across studies.

Effect measures were more consistently reported for biological determinants than for psychological, behavioral, and social determinants. Studies in the latter domains frequently reported directional associations, qualitative findings, p-values without comparable effect measures, or estimates based on different outcome definitions and reference categories. These findings were therefore synthesized narratively according to the direction and clinical context of the association, while numerical estimates were reported only when they were available and sufficiently interpretable (**Table 2**).

### 3.3 Annual scientific production

The annual scientific production on biopsychosocial determinants of TB care and treatment outcomes increased over the study period (**Figure 2**). Based on metadata retrieved from PubMed, publication output remained relatively low during the early years, followed by a gradual increase throughout the 1990s and 2000s. Annual production increased more rapidly from the early 2010s, approaching 200 documents by the late 2010s and remaining above this level in most subsequent years. The highest annual output was recorded in 2025 (279 documents). The lower number of publications in 2026 reflects the incomplete coverage of that publication year at the time of data retrieval.

**Figure 2:**
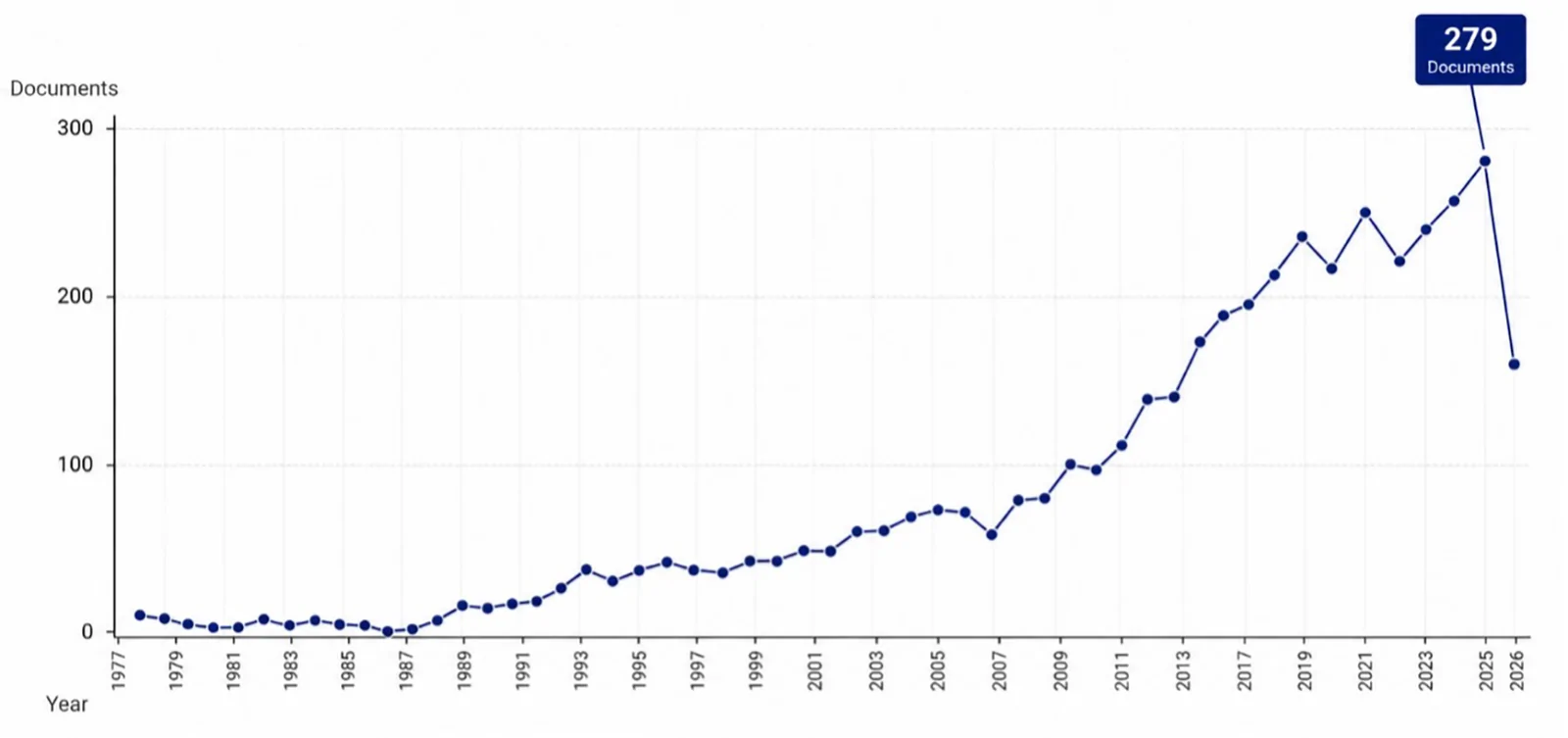
Annual scientific production of pulmonary tuberculosis research. Annual publication output of the 3,350 articles retrieved from PubMed and analysed using Biblioshiny, the web interface of the Bibliometrix R package. The number of publications increased progressively over the study period, with a pronounced acceleration from the early 2010s onwards and a peak of 279 Documants in 2025. The lower publication count for 2026 reflects an incomplete publication year at the time of data retrieval.

### 3.4 Country scientific production

The geographical distribution of scientific production demonstrated broad global participation in research on biopsychosocial determinants of TB care and treatment outcomes (**Figure 3**). Publications originated from countries across North America, South America, Europe, Africa, Asia, and Oceania, although research output varied considerably between countries. The United States and India produced the largest number of publications, followed by China, which contributed 1,904 documents. Other countries showed lower but variable publication outputs, indicating broad international participation in this research field.

**Figure 3:**
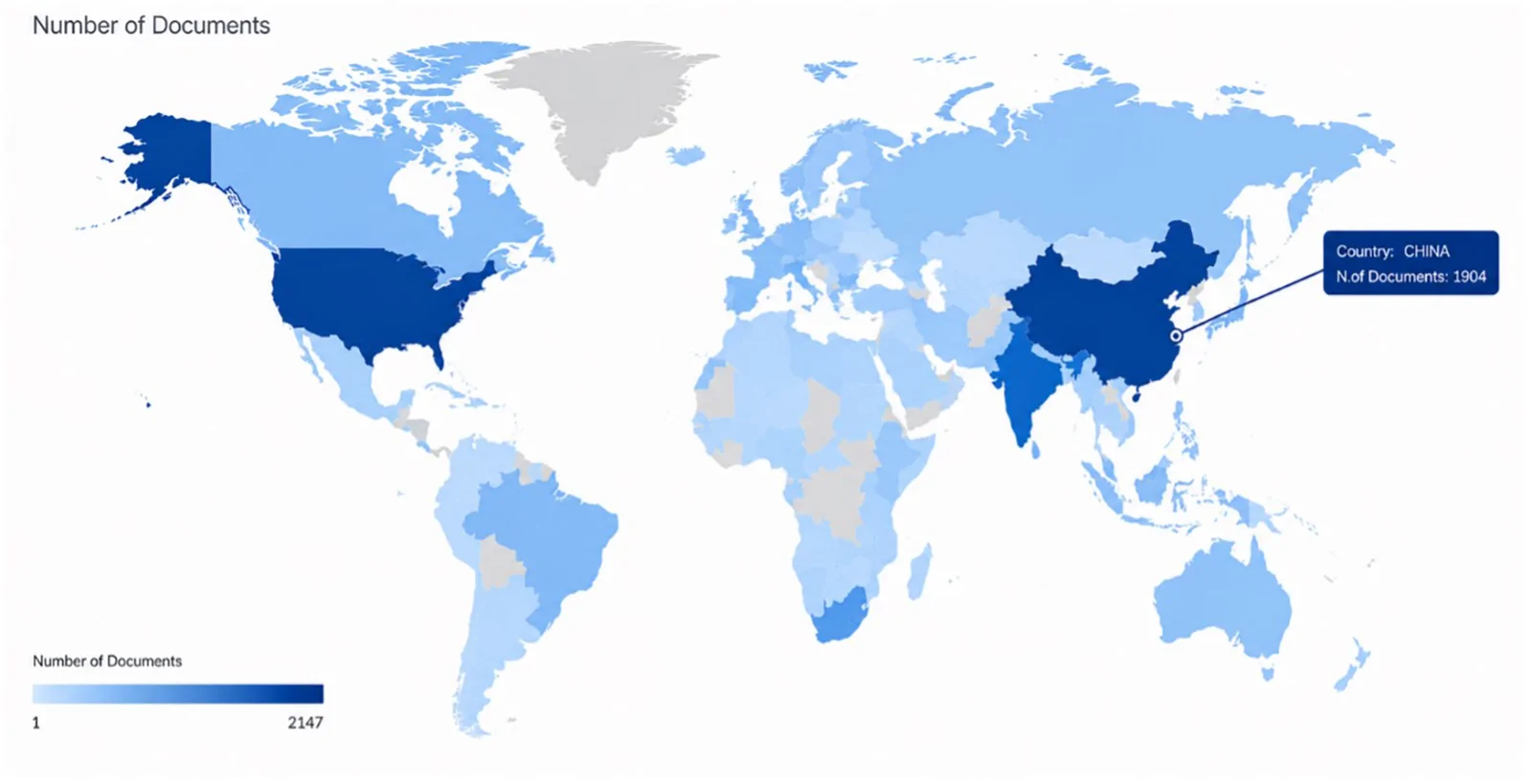
Country scientific production in biopsychosocial and pulmonary tuberculosis research. Geographical distribution of scientific production among the 3,350 articles retrieved from PubMed and analyzed using Biblioshiny, the web interface of the Bibliometrix R package. Countries are shaded according to their publication output, with darker shades representing higher numbers of documents. The United States and India showed the highest scientific production, followed by China, which contributed 1,904 documents.

### 3.5 Temporal evolution co-authorship networks

The overlay visualization of the co-authorship network analyzed by VOSviewer identified two major collaborative structures among authors publishing on biopsychosocial determinants of TB care and treatment outcomes (**Figure 4**). Node colors represent the average publication year, ranging from earlier publications (blue and purple) to more recent publications (green and yellow).

**Figure 4:**
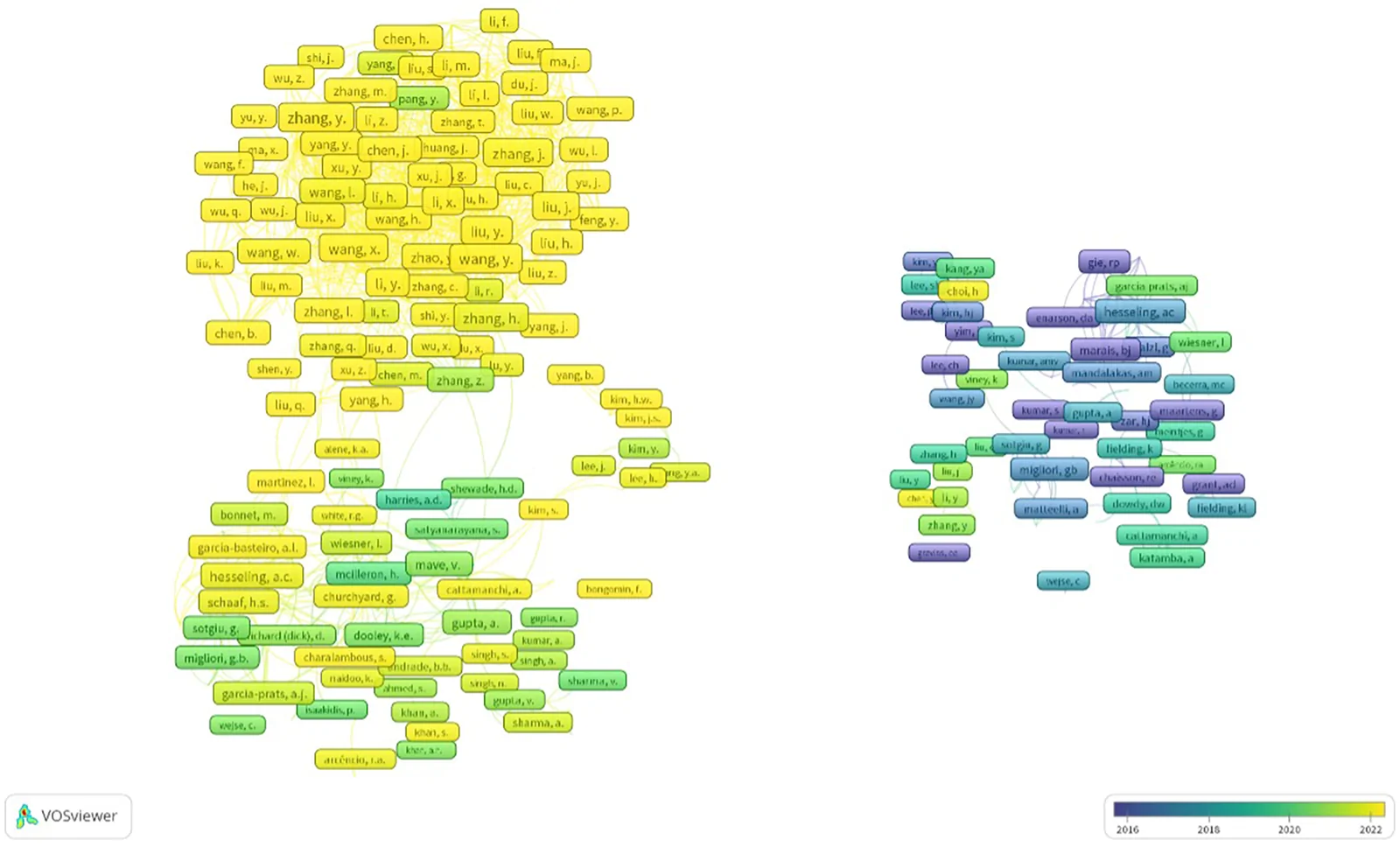
Overlay visualization of the co-authorship network in biopsychosocial and pulmonary tuberculosis research. Each node represents an author, and links indicate co-authorship relationships. Node colors represent the average publication year, ranging from earlier activity in blue and purple to more recent activity in green and yellow. The network highlights established international collaborative groups and the recent expansion of a densely interconnected Chinese author community. The visualization was generated using VOSviewer.

The first major collaborative structure comprised a densely connected group of predominantly Chinese authors, including Zhang, Wang, Liu, Li, Chen, Yang, and Wu. Most nodes within this cluster were yellow, indicating relatively recent publication activity. A second group within the same structure included Hesseling AC, Garcia-Prats AJ, Wiesner L, Churchyard G, Dooley KE, Gupta A, Mave V, Migliori GB, and Sotgiu G. These authors were represented mainly by green-to-yellow nodes and showed connections with the larger Chinese collaboration network. The second major collaborative structure was characterized of blue, purple, and turquoise nodes, indicating comparatively earlier publication years. This cluster included authors such as Marais BJ, Mandalakas AM, Chaisson RE, Grant AD, Fielding K, and Cattamanchi A. Connections between the two major collaborative structures were limited.

### 3.6 Thematic structure of TB care and treatment outcomes research

**Figure 5** presents the author keyword co-occurrence network generated using VOSviewer. Node size represents keyword frequency, link thickness represents co-occurrence strength, and node color represents thematic clusters identified by the VOSviewer clustering algorithm.

**Figure 5:**
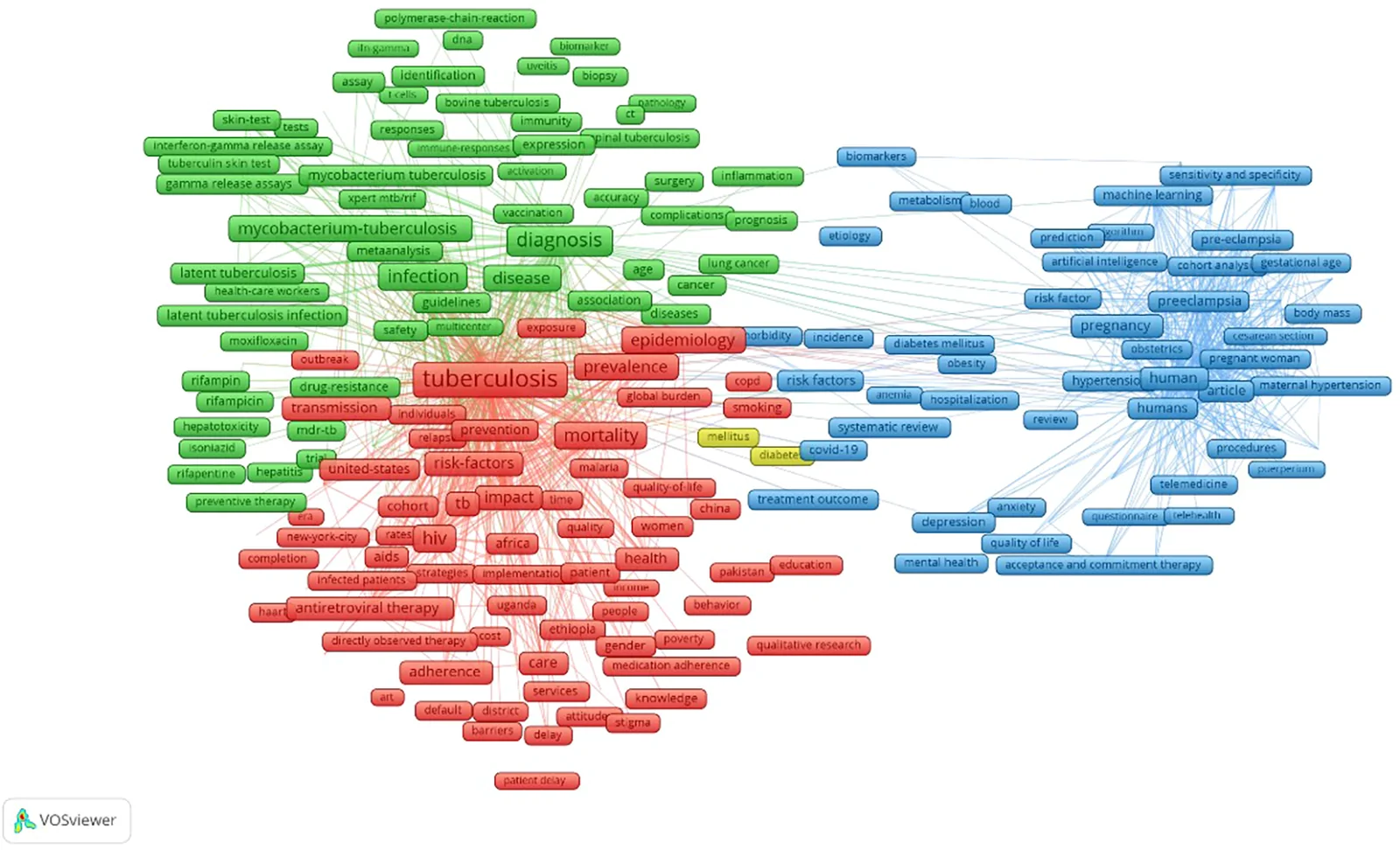
Network visualization of author keyword co-occurrence generated using VOSviewer. Node size represents keyword frequency, line thickness indicates the strength of co-occurrence, and colors denote thematic clusters. The network demonstrates strong interconnections among biological and genetic research (e.g., Mycobacterium tuberculosis, DNA, PCR, biomarkers, drug resistance), clinical research (e.g., diagnosis, epidemiology, treatment outcome, machine learning), and biopsychosocial determinants (e.g., adherence, mental health, depression, quality of life, stigma, barriers, and health services). The close integration of these domains highlights the evolution of biopsychosocial and TB care and treatment outcomes toward a comprehensive biopsychosocial-genetic framework, emphasizing that disease outcomes are influenced by interactions among molecular mechanisms, clinical characteristics, psychological well-being, and social determinants of health.

One major cluster comprised keywords related to psychological and behavioral aspects of TB, including adherence, medication adherence, patient delay, stigma, barriers, behavior, education, care, quality of life, mental health, depression, anxiety, acceptance and commitment therapy, and telemedicine. A second cluster included social and health-service-related keywords such as HIV, Africa, poverty, health, care, services, education, stigma, barriers, adherence, patient delay, and directly observed therapy. The biological and genetic cluster contained keywords including Mycobacterium tuberculosis, diagnosis, infection, expression, DNA, polymerase chain reaction (PCR), biomarker, immune response, interferon-gamma release assay, Xpert MTB/RIF, drug resistance, rifampicin, isoniazid, and MDR-TB. Clinical research keywords, including diagnosis, epidemiology, prevalence, mortality, risk factors, treatment outcome, cohort, and comorbidity, occupied central positions within the network. Additional keywords included machine learning, artificial intelligence, prediction, and biomarkers.

### 3.7 Temporal evolution of research themes

**Figure 6** presents the temporal overlay visualization of author keyword co-occurrence over the past 10 years. Node size represents keyword frequency, links indicate co-occurrence relationships, and node colors represent the average publication year. Earlier research topics are shown in blue and purple, whereas more recent topics are shown in green and yellow.

**Figure 6:**
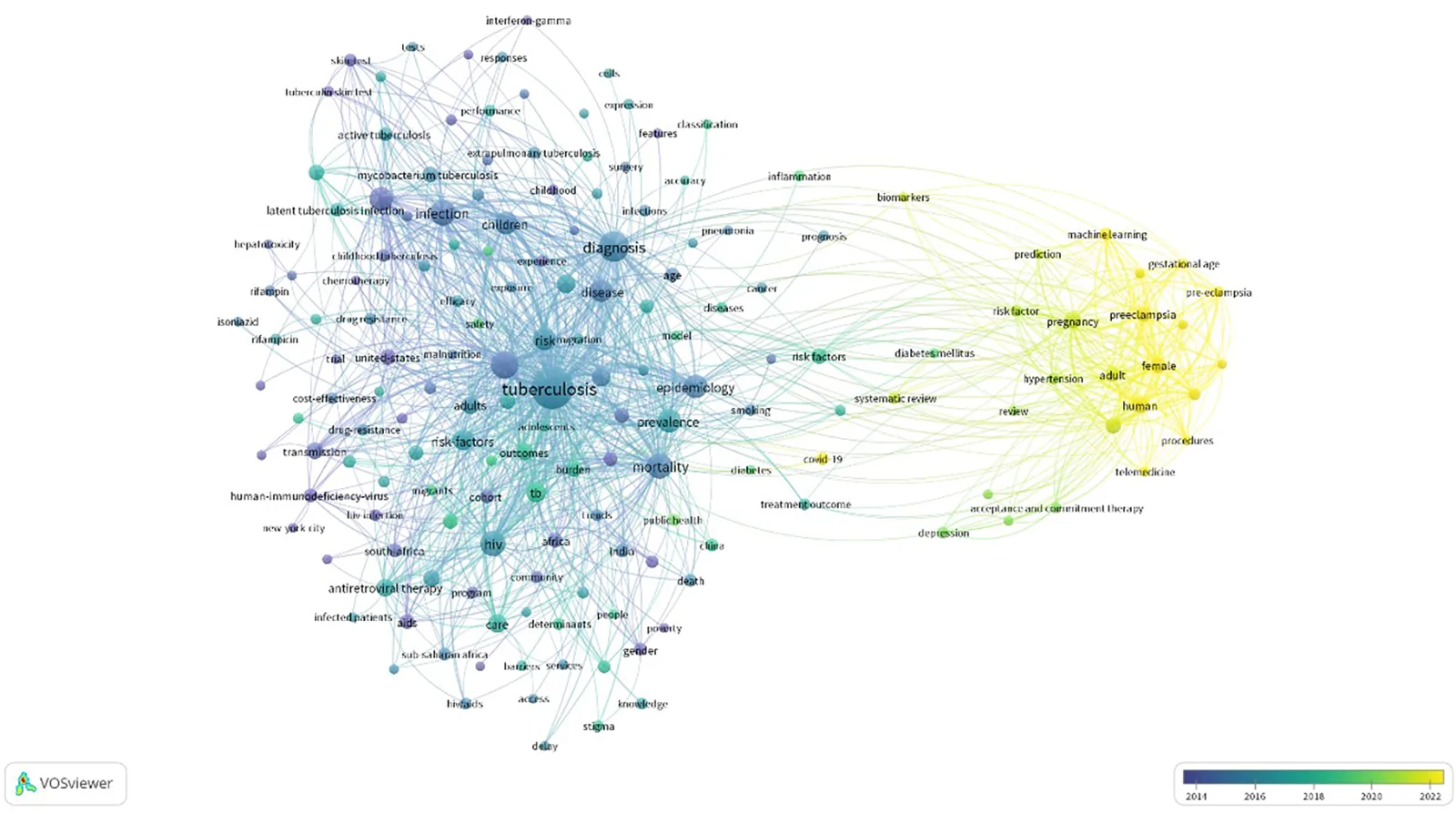
Overlay visualization of author keyword co-occurrence over the past 10 years. The visualization illustrates the temporal evolution of research themes based on author keyword co-occurrence. Node size represents the frequency of keyword occurrence, links indicate co-occurrence relationships between keywords, and node color represents the average publication year, ranging from earlier topics in blue and purple to more recent topics in green and yellow. The network demonstrates a temporal progression from established themes related to tuberculosis, diagnosis, infection, epidemiology, and HIV toward emerging topics involving comorbidities, mental health, pregnancy-related conditions, biomarkers, prediction, telemedicine, and machine learning.

Earlier research focused primarily on tuberculosis diagnosis, infection, treatment, childhood TB, latent tuberculosis infection, active tuberculosis, drug resistance, rifampicin, isoniazid, chemotherapy, and clinical trials. Keywords related to HIV, AIDS, antiretroviral therapy, mortality, epidemiology, prevalence, and sub-Saharan Africa were also prominent during the earlier-to-intermediate period. Geographical keywords included Africa, South Africa, India, China, and the United States. During the intermediate period, frequently occurring keywords included risk factors, epidemiology, prevalence, mortality, cohort, outcomes, burden, migrants, community, care, services, access, barriers, stigma, poverty, and gender. Keywords related to diabetes, hypertension, smoking, cancer, inflammation, prognosis, treatment outcome, and COVID-19 appeared predominantly during the intermediate-to-recent period. More recent publications included keywords such as biomarkers, prediction, machine learning, pregnancy, preeclampsia, gestational age, female, hypertension, depression, acceptance and commitment therapy, and telemedicine.

### 3.8 Integrated synthesis of scoping and bibliometric findings

Comparison of the scoping review and bibliometric findings showed overlap between frequently reported determinants and the dominant research themes identified in the author keyword network (**Figure 6**). HIV co-infection, smoking, alcohol use, diabetes, treatment adherence, stigma, limited TB knowledge, poverty, and barriers to healthcare access were represented in both analyses. Earlier research focused primarily on HIV, infection, diagnosis, and drug resistance, whereas healthcare access, comorbidities, mental health, and patient-centered care appeared more frequently during the intermediate and recent periods.

Several determinants that were frequently associated with adverse TB outcomes in the scoping review, including malnutrition or low BMI, distance to healthcare facilities, social and family support, homelessness, and migration, were less prominent within the keyword network. In contrast, biomarkers, prediction, machine learning, and telemedicine featured prominently in the bibliometric analysis but were less frequently represented in the empirical evidence synthesized in the scoping review.

## 4 Discussion

This review provides a comprehensive overview of the biological, psychological, and social determinants associated with TB care and treatment outcomes by integrating a scoping review with bibliometric analysis. Across 78 identified determinants, HIV co-infection, smoking, alcohol use, diabetes, malnutrition, stigma, limited TB knowledge, poverty, and barriers to healthcare access emerged as the factors most consistently associated with delayed diagnosis, poor treatment adherence, unsuccessful treatment outcomes, multidrug-resistant TB (MDR-TB), and mortality. In parallel, the bibliometric analysis demonstrated that the research field has evolved from a predominantly biomedical focus towards broader consideration of mental health, social determinants, health services, and digital health. By integrating evidence mapping with bibliometric analysis, this review not only synthesizes the determinants associated with TB care and treatment outcomes but also characterizes the evolution of the research landscape, identifies evidence gaps, and highlights emerging research priorities. Compared with previous reviews that have focused on individual determinants or specific stages of TB care, this integrated approach provides a broader perspective on the current evidence base and its implications for future multidisciplinary research and person-centered TB care (Munro et al., 2007; Courtwright and Turner, 2010; Hargreaves et al., 2011; Craig et al., 2017).

The findings confirm that biological determinants remain fundamental predictors of TB outcomes. HIV co-infection was consistently associated with mortality, MDR-TB, and unsuccessful treatment outcomes, while diabetes, malnutrition, low BMI, older age, and severe clinical presentation were also repeatedly associated with adverse outcomes. These findings are consistent with previous epidemiological studies and WHO reports demonstrating that impaired immune function and metabolic comorbidities substantially increase susceptibility to disease progression and poor treatment outcomes (Jeon and Murray, 2008; Getahun et al., 2010; Baker et al., 2011). However, biological vulnerability rarely occurs in isolation. Individuals with diabetes or malnutrition frequently experience concurrent socioeconomic disadvantage, barriers to healthcare access, and psychological distress, which may collectively influence diagnosis, treatment initiation, adherence, and long-term outcomes (Lönnroth et al., 2009; Hargreaves et al., 2011). These findings support the value of interpreting biological risk within a broader biopsychosocial context (Engel, 1977).

Psychological and social determinants were consistently associated with multiple stages of the TB care continuum. Smoking, alcohol use, stigma, limited TB knowledge, poor social support, migration, poverty, homelessness, and geographical barriers to healthcare were repeatedly linked to diagnostic delay, treatment interruption, loss to follow-up, and unsuccessful treatment outcomes (Bates et al., 2007; Munro et al., 2007; Lönnroth et al., 2008; Storla et al., 2008; Courtwright and Turner, 2010; Hargreaves et al., 2011). These findings reinforce the growing recognition that successful TB control depends not only on effective antimicrobial therapy but also on addressing the social and behavioral barriers that influence access to and retention in care. Many of these determinants are potentially modifiable through health education, psychological support, nutritional assistance, transport support, community-based treatment program, and social protection policies (Munro et al., 2007; Lönnroth et al., 2009; Hargreaves et al., 2011; Volmink and Garner, 2026; Alipanah et al., 2018). Although the specific implementation of these interventions should be adapted to local contexts, their consistent association with TB outcomes across diverse settings suggests broad relevance for TB programs.

Although malnutrition, inadequate social support, migration, homelessness, and geographical access have received comparatively limited research attention, the available evidence indicates clinically and programmatically important consequences. Malnutrition was examined in only four studies but was associated with unsuccessful MDR-TB treatment (aOR = 2.99) and pediatric mortality (OR = 2.98). Travel distances exceeding 5 km were repeatedly associated with delayed treatment initiation and non-adherence, whereas the presence of family or treatment support was associated with improved adherence and treatment success. Migrants and people experiencing homelessness may face overlapping vulnerabilities, including residential instability, financial insecurity, stigma, mobility, and fragmented continuity of care, which can delay diagnosis and interrupt treatment. By reducing timely case detection, treatment completion, and continuity of follow-up, these determinants may sustain transmission and impede progress toward TB elimination even when effective antimicrobial treatment is available (Munro et al., 2007; Lönnroth et al., 2009; Hargreaves et al., 2011; Volmink and Garner, 2026; Alipanah et al., 2018; **Table 2**).

The bibliometric analysis demonstrates a clear evolution in the intellectual structure of TB research. Earlier research predominantly focused on microbiology, diagnosis, antimicrobial therapy, drug resistance, and TB–HIV co-infection. Over time, research expanded to include epidemiology, healthcare access, stigma, behavioral determinants, quality of life, and more recently biomarkers, machine learning, telemedicine, and precision medicine. This evolution reflects increasing recognition that TB management extends beyond pathogen control to encompass person-centered care and health system performance (Uplekar et al., 2015; Subbaraman et al., 2018; Lee et al., 2020; Donthu et al., 2021; Qin et al., 2021). Although emerging topics such as digital health and artificial intelligence have gained increasing research attention, they currently represent a smaller proportion of the empirical evidence on determinants associated with TB care and treatment outcomes than established biological, behavioral, and social determinants (Subbaraman et al., 2018; Lee et al., 2020; Qin et al., 2021).

An important contribution of this review is the integration of evidence mapping with science mapping. Several determinants that were consistently associated with adverse TB outcomes in the scoping review, including malnutrition, social support, migration, homelessness, and geographical access to healthcare, were less prominent within the bibliometric keyword network. Conversely, machine learning, biomarkers, prediction, and telemedicine have emerged as increasingly visible research themes despite comparatively limited evidence directly linking these approaches to patient-centered outcomes (Hargreaves et al., 2011; Subbaraman et al., 2018; Lee et al., 2020; Donthu et al., 2021; Qin et al., 2021). This discrepancy suggests that research activity does not always correspond to determinants with the greatest public health relevance. Digital health and machine-learning approaches may improve case detection, risk stratification, treatment monitoring, and continuity of care. However, their benefits may be constrained by unequal access to digital devices and connectivity, limited digital literacy, incomplete routine data, and the underrepresentation of marginalized populations in data used to develop or validate algorithms. These technologies should therefore be evaluated not only for predictive performance, but also for accessibility, acceptability, equity, and their capacity to complement rather than replace interventions addressing poverty, stigma, migration, homelessness, and geographical barriers to care (Subbaraman et al., 2018; Lee et al., 2020; Qin et al., 2021).

The findings have several implications for research, policy, and practice. Future research should prioritize designs capable of clarifying causal pathways and evaluating intervention effects. Prospective longitudinal cohort studies are needed to assess how nutritional, psychological, and social vulnerabilities influence diagnosis, adherence, recurrence, and mortality over time. Mixed-methods studies should examine care pathways and contextual mechanisms among migrants, people experiencing homelessness, incarcerated populations, children, rural communities, and individuals with MDR-TB. Pragmatic or cluster-randomized trials should evaluate interventions such as nutritional supplementation, transport assistance, cash or social-protection support, family or peer treatment support, and community-based adherence programs. Implementation studies in high-burden, resource-constrained settings should assess not only effectiveness but also reach, acceptability, feasibility, equity, and sustainability. Future studies should also apply harmonized definitions of diagnostic delay, treatment interruption, loss to follow-up, treatment success, recurrence, and mortality to improve comparability across settings (Lönnroth et al., 2009; Hargreaves et al., 2011; Peters et al., 2013; Alipanah et al., 2018).

This review has several strengths. By integrating a scoping review with bibliometric analysis, it provides complementary insights into both the empirical evidence and the evolution of the research landscape (Arksey and O’Malley, 2005; Peters et al., 2015; Donthu et al., 2021). The comprehensive search across multiple international databases enabled identification of a broad range of biological, psychological, and social determinants across the TB care continuum, while the integrated synthesis facilitated comparison between the available evidence and the development of research priorities.

Several limitations should also be considered. Only peer-reviewed articles published in English were included, which may have excluded relevant evidence reported in other languages or grey literature (Paez, 2017). This restriction may have introduced language and publication biases and may have underrepresented locally produced, programmatic, community-based, and policy evidence, particularly from high-burden, resource-constrained settings. Consequently, the relative prominence of determinants and the identified evidence gaps may not fully reflect local priorities or implementation realities. Although the included evidence covered all WHO regions, differences in health-system capacity, social protection, diagnostic access, digital infrastructure, and data availability limit direct generalization across settings; the findings should therefore be interpreted and applied in relation to local epidemiological and service-delivery contexts. The substantial heterogeneity in study designs, populations, outcome definitions, and effect measures precluded quantitative synthesis (Tricco et al., 2018; Peters et al., 2020). In addition, bibliometric analyses describe publication patterns rather than study quality and may be influenced by database coverage and citation practices (van Eck and Waltman, 2010; Donthu et al., 2021). Finally, as this review synthesized observational evidence, the identified relationships should be interpreted as associations rather than evidence of causality (Grimes and Schulz, 2002).

## 5 Conclusion

In summary, this integrated scoping review and bibliometric analysis identified HIV co-infection, diabetes, malnutrition, smoking, alcohol use, stigma, limited TB knowledge, poverty, and barriers to healthcare access as determinants consistently associated with adverse outcomes across the TB care continuum. Bibliometric findings showed a shift from predominantly biomedical topics toward mental health, social determinants, digital health, and data-driven approaches, while also revealing a mismatch between emerging research trends and determinants with strong empirical relevance. These findings support integrated TB-control strategies that address biological, psychological, social, and structural determinants and prioritize under-researched factors across diverse settings.

## Data Availability

The data analyzed in this study is subject to the following licenses/restrictions: Data available upon request. Requests to access these datasets should be directed to bayu.satria@ugm.ac.id.
